# Genistein inhibits estradiol- and environmental endocrine disruptor-induced growth effects on neuroblastoma cells *in vitro*

**DOI:** 10.3892/ol.2013.1236

**Published:** 2013-03-07

**Authors:** JICUI ZHENG, HUI LI, HAITAO ZHU, XIANMIN XIAO, YANGYANG MA

**Affiliations:** 1 Department of Pediatric Surgery, Children’s Hospital, Fudan University, Shanghai 201102;; 2 Department of Pediatric Surgery, Children’s Hospital, Jiao Tong University, Shanghai 200040, P.R. China

**Keywords:** neuroblastoma, environmental endocrine disruptors, genistein, Akt

## Abstract

The aim of this study was to examine the effect of genistein on human neuroblastoma cell proliferation induced by two common environmental endocrine disruptors, bisphenol A (BPA) and Di-2-ethylhexyl phthalate (DEHP), and to investigate its underlying mechanism. SK-N-SH human neuroblastoma cells were treated with E_2_ (1 ng/ml), BPA (2 *μ*g/ml) or DEHP (100 *μ*M), with or without genistein (12.5 *μ*M) *in vitro*. The number of viable cells was detected with an absorbance reader after 0, 24, 48 or 72 h treatment. The percentage of cells in different phases, and expression of Akt and its phosphorylation levels were also assessed by flow cytometry and western blot analysis at 72 h, respectively. The BPA and DEHP groups had a 30% higher number of viable cells compared to the non-treated group at 48 h (P<0.001). However, the cell numbers did not increase significantly in the groups with additional treatment with genistein (P>0.05 vs. control) and the same trend was observed at 72 h. The expression of phospho-Akt protein was increased in the groups treated with BPA or DEHP compared to the control group at 72 h (P<0.05), while no significant elevation in the expression of phospho-Akt was observed (P>0.05) in genistein-treated groups. Cells were arrested at the G_2_/M phase by genistein. Similar effects were observed in the E_2_ group with or without genistein treatment. Akt protein expression had no significant change among all the groups (P>0.05). In conclusion, estradiol- or environmental endocrine disruptor-induced proliferation of human neuroblastoma cells is effectively abolished by genistein, likely in a cell cycle- and Akt pathway-dependent manner.

## Introduction

Neuroblastoma is a common extracranial childhood solid tumor (1,2). The majority of neuroblastomas are highly metastastic with poor clinical outcome despite the application of intensive multimodal therapy (3). However, its oncogenesis is still poorly understood. Our previous studies showed that environmental endocrine disruptors (EEDs) may be one of the most important causes of the carcinogenesis of neuroblastoma (4,5). A recent multicenter case-control survey also showed that paternal exposure to hydrocarbons was associated with an increased incidence of neuroblastoma in children (6). Therefore, specific antagonists blocking EED-dependent adverse effects may potentially protect against neuroblastoma occurrence and become a new option for the treatment of patients with neuroblastoma in the clinical practice.

Isoflavones are natural compounds that commonly exist in soy-based foods. They display a variety of biological activities including suppression of activity of several enzymes that regulate cell proliferation (7), prevention of cell-cycle progression (8) and inhibition of tumor growth (7). Genistein 4′,5,7-trihydroxyisoflavone, also known as genistein, is one of the major soy isoflavones. Genistein is considered to be a promising therapeutic candidate for various cancers (9–11).

However, the effects of genistein on neuroblastoma cell growth, especially those induced by EED and its molecular mechanisms, are rarely reported. In this study, we used SK-N-SH human neuroblastoma cells as a cell model, and investigated the effect of genistein on the bisphenol A (BPA)-and Di-2-ethylhexl phthalate (DEHP)-induced proliferation of human neuroblastoma *in vitro*.

## Materials and methods

### Cell line, reagents and antibodies

SK-N-SH, a human neuroblastoma cell line, was purchased from Shanghai Institute for Biological Sciences, Chinese Academy of Sciences (Shanghai, China). Roswell Park Memorial Institute-1640 (RPMI-1640) medium, phenol red-free RPMI-1640 medium and fetal bovine serum (FBS) were obtained from Gibco (Invitrogen, Carlsbad, CA, USA). 17β-estradiol (E_2_), genistein, sulphatase and dextran-coated charcoal were purchased from Sigma (St. Louis, MO, USA). Cell counting kit-8 (CCK-8) was obtained from Dojindo Laboratory (Kumamoto, Japan). BPA and DEHP were purchased from Shanghai Chemical Reagents Company (Shanghai, China). Akt and phospho-Akt (Ser473; p-Akt) antibodies were obtained from Cell Signaling Technology (Beverly, MA, USA). E_2_, BPA, DEHP and genistein were dissolved in Dimethyl Sulfoxide (DMSO), and then diluted with phenol red-free RPMI-1640 medium containing charcoal-dextran-stripped FBS (cd-FBS) to final concentration for culturing at 1 ng/ml E_2_, 2 *μ*g/ml BPA, 100 *μ*M/l DEHP and 12.5 *μ*M/l genistein, respectively. The final solvent concentration in the culture did not exceed 0.1%.

### Cell culture and treatment

SK-N-SH cells were cultured in 75-cm^2^ flasks with RPMI-1640 medium supplemented with 0.1 M L-glutamine, 10% (v/v) FBS, 100 U/ml of penicillin, 100 *μ*g/ml of streptomycin at 37°C with 5% CO2 in a fully humidified incubator. Prior to treatments, cells were starved in phenol red-free RPMI-1640 medium without FBS for 24 h. The study was approved by the ethics committee of Children’s Hospital, Fudan University.

### CCK-8 assay

SK-N-SH cells were seeded into 96-well flat-bottomed microtiter plates at a density of 10^5^/ml and the final volume in each well was 100 *μ*l. After being starved, cells were then treated with 1 ng/ml E_2_, 2 *μ*g/ml BPA, or 100 *μ*M/l DEHP with or without 12.5 *μ*M/l genistein. The number of viable cells was detected with CCK-8 assay as described previously, at 0, 24, 48 and 72 h (4,5). The treatment wells were in quadruplicates and the whole experiment was repeated independently three times.

### Flow cytometry

Cells were cultured into 6-well plates at a density of 3×10^5^/ml, and then treated as described above. After 72 h incubation, 10^6^ cells were fixed with 1 ml 70% ethanol at 4°C for 30 min and then stained with staining solution (2 mg/ml propidium iodide and 10 mg/ml RNase in PBS) at 37°C for 30 min. Stained cells were analyzed for fluorescence intensity with a fluorescence-activated cell sorter (Becton Dickinson, San Jose, CA, USA) equipped with an argon laser emitting at 488 nm, using the CellQuest software (Becton Dickinson). A minimum of 10,000 events were acquired for each determination. The percentages of cells in G_2_/M phases were calculated by the ModFit program (Becton Dickinson).

### Western blot analysis

Neuroblastoma cells were plated in 100-mm dishes and subsequently treated as described above for 72 h. Then cells were collected in RIPA buffer (50 mmol/l Tris-HCl, pH 7.5, 150 mmol/l NaCl, 0.5% deoxycholate, 0.1% sodium dodecyl sulfate) on ice for 15 min. Protein concentrations were determined by the Bradford method and protein extracts (50 *μ*g/lane) were loaded on an 8% SDS-polyacrylamide gels. After gel separation, proteins were transferred to a nitrocellulose membrane. The membrane was blocked with 5% skimmed milk, then separately incubated with anti-Akt antibody (1:1000 dilution), anti-p-Akt antibody (1:200 dilution). Incubation with anti-β-actin antibody (1:5000 dilution) was performed together as an internal control. The membranes were visualized with an enhanced chemiluminescence system according to the manufacturer’s instructions. The bands on the western blot films were scanned by VersaDoc Image Analysis System (Bio-Rad, Hercules, CA, USA), and analyzed with the QualityOne Image Analysis software (Bio-Rad).

### Statistical analysis

All results were analyzed using SPSS 11.5 software (SPSS Inc., Chicago, IL, USA). Data were expressed as mean ± standard error of mean (SEM) of separate experiments (n≥3) and compared by one-way analysis of variance (ANOVA). The difference between two treatments was considered significant at P<0.05.

## Results

### Genistein suppressed SK-N-SH cell proliferation induced by E_2_, BPA and DEHP

**Figure 1:**
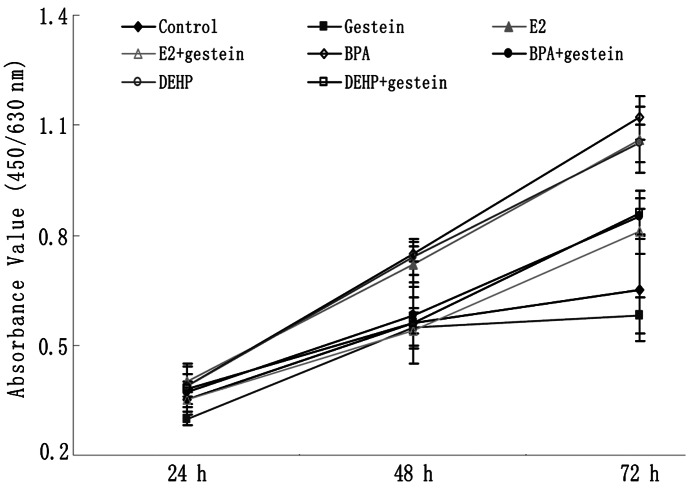
Growth curves of SK-N-SH cells from 0 to 72 h after drug treatment. SK-N-SH cells were treated with 1 ng/ml 17β-estradiol (E_2_), 2 *μ*g/ml bisphenol A (BPA) or 100 μM/l di-2-ethylhexl phthalate (DEHP), with or without 12.5 *μ*M/l genistein. BPA, DEHP and E_2_ significantly promoted the proliferation of SK-N-SH cells *in vitro* at 48 and 72 h, while genistein inhibited the proliferation effects.

The effect of genistein on the proliferation of neuroblastoma cells was assessed using CCK-8 proliferation assay. Time- and dose-dependent experiments showed that genistein inhibited the SK-N-SH cell growth in a dose-dependent manner (data not shown). After 24 h treatment, absorbance values (AVs) among all the groups showed no significant difference. Then at 48 h, the AVs of the E_2_, BPA and DEHP groups (0.72±0.06, 0.75±0.02, 0.74±0.05, respectively) were significantly higher than those of the control group (0.56±0.03, P<0.001; Fig. 1). However, there was not a similar increase of AV in groups treated with E_2_, BPA or DEHP with additional genistein treatment. The AVs in these groups were not significantly higher than those in the control group (P>0.05), but were evidently lower than those in the groups treated with E_2_, BPA and DEHP alone (P<0.001; Fig. 1). At 72 h, a similar phenomenon was noted (Fig. 1).

### Genistein arrested SK-N-SH cells at G_2_/M phase of cell cycle

After 72 h treatment, flow cytometric analysis was also applied to investigate whether the effect of genistein inhibition against neuroblastoma cell proliferation occurred in a cell cycle-dependent manner. When cells were treated with additional genistein, the percentage of cells in the G_2_/M phase significantly increased. Cells were arrested at the G_2_/M phase of the cell cycle (P>0.05 vs. the control group; P<0.01 vs. E_2_, BPA or DEHP groups; Table I).

### Genistein modulated p-Akt (Ser473) protein expression

**Figure 2:**
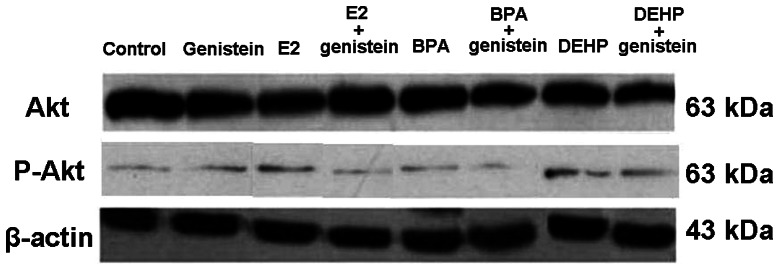
The expression of Akt and p-Akt in SK-N-SH cells detected by western blot analysis. At 72 h, the expression of Akt was abundant in every group. p-Akt was abundant in the E2, BPA and DEHP group, while weak in the control and genistein group. BPA, bisphenol A; DEHP, Di-2-ethylhexyl phthalate.

Akt and p-Akt expression levels were further detected by western blot analysis to determine the principal signaling mechanism of genistein inhibiting SK-N-SH cell growth at 72 h after drug treatments. The expression of p-Akt was abundant when treated with E_2_, BPA or DEHP only (Fig. 2). By contrast, there was no increase in the expression of p-Akt protein in the genistein-treated groups (Fig. 2). Akt protein expression had no significant change in any of the groups.

## Discussion

During the last decade, soy isoflavones mainly derived from soybean received much attention as dietary components having inhibitory effects on cancers. The lower risk of breast and prostate cancers in Asians, who consume 20–50 times more soy than Americans, has raised the question whether compounds in the soy diet act as a natural chemopreventive agent. Indeed, a cross-national study involving 59 countries identified that soy products have a highly significant effect against the development of prostate cancer (12). Elevated levels of soy isoflavones in the micromolar range have been detected in the serum, urine, prostatic fluid and prostate tissue in vegetarians and Asian men who consume a soy-rich diet and have low risk of prostate cancer (13). In contrast, serum concentration of genistein in Americans and Europeans is in the nanomolar range (13).

Soy isoflavones include genistein, daidzein and glycitein. However, genistein is the principal isoflavone in soy that has been demonstrated to be responsible for reducing the incidence of hormone-related cancers. In laboratory *in vitro* experiments, genistein has been found to inhibit the growth of various cancer cell lines including prostate and breast cancer cells (14). Moreover, the evidence from *in vitro* studies has demonstrated that genistein exerts its inhibitory effects on the development of cancers, cancer cell growth, cancer progression, cancer cell invasion, metastasis and angiogenesis (14).

The direct anti-tumor activity, action mechanisms and therapeutic potential of genistein on neuroblastoma cells have been investigated (8,15). We have previously reported that environmental endocrine disruptor BPA promoted the proliferation of SK-N-SH cells *in vitro* and *in vivo*(4,5). However, the effects of genistein on the growth-promoted action of BPA and DEHP on human neuroblastoma cells and its action mechanisms remain poorly understood. In the current study, we hypothesized that genistein may also inhibit the human neuroblastoma cell proliferation induced by EED and estrodiol *in vitro*.

Our results showed that the proliferation of neuroblastoma cells is enhanced by estrogen and certain environmental estrogen-like contaminants. When genistein was used in combination with E_2_, BPA or DEHP, cell growth was inhibited effectively. This is in agreement with other studies involving several types of cells (16–18). The present results demonstrated that genistein suppressed SK-N-SH cell proliferation induced by BPA and DEHP.

In addition, genistein prevented the SK-N-SH cells from entering the G_2_/M phase during the cell cycle, suggesting that the observed growth-inhibitory effect of genistein was mediated through modulation of cell cycle progression in SK-N-SH cells. Our results are in line with others which showed that genistein exerts its anti-tumor effects by arresting SK-N-SH cells in G_2_/M phase and inhibiting cancer cell death (19).

The phosphatidylinositol-3 kinase/Akt (PI3K/Akt) signaling pathway plays a critical role in cell survival and apoptosis. Inhibition of the PI3K/Akt pathway has been considered as a therapeutic target for cancer where PI3K/Akt activation is a causative factor. It has also been reported that genistein inhibits the activation of the Akt signaling pathway in prostate and breast cancer cell lines (20,21). In our study, the expression of p-Akt was abundant in the E_2_, BPA and DEHP groups, but genistein caused a decrease in active p-Akt levels.

In summary, BPA and DEHP have growth-promoting effects on human neuroblastoma SK-N-SH cells *in vitro*, which can be inhibited by genistein. Genistein significantly inhibited the growth of neuroblastoma cells through modulation of cell cycle progression and the PI3K/Akt pathway. The results of our study provide the experimental basis for the application of genistein in additional preventive or therapeutic strategies in neuroblastoma.

## Figures and Tables

**Table I:** G_2_/M phase analysis of SK-N-SH cells at 72 h.

Groups	G_2_/M (%)	Groups	G_2_/M (%)
Control	9.15±1.08	Genistein	16.58±2.71^c^
E_2_	11.70±1.14^a^	E2+genistein	15.56±0.58^b^
BPA	11.94±0.06^a^	BPA+genistein	17.76±1.45^c^
DEHP	11.64±0.39^a^	DEHP+genistein	15.99±0.98^c^

Compared with control group,

a P<0.05; Compared with groups without genistein,

b P<0.01;

c P<0.001. BPA, bisphenol A; DEHP, Di-2-ethylhexyl phthalate.
